# Development and Validation of the Questionnaire for Adaptive Hyperactivity and Goal Achievement (AHGA)

**DOI:** 10.2174/17450179-v19-e230419-2022-50

**Published:** 2023-05-12

**Authors:** Goce Kalcev, Giulia Cossu, Antonio Preti, Maria Teresa Littera, Stèphanie Frau, Diego Primavera, Rosanna Zaccheddu, Veronica Matza, Marta Ermellino, Elisa Pintus, Mauro G. Carta

**Affiliations:** 1 Department of Innovation Sciences and Technologies at the University of Cagliari, Cagliari, Italy; 2 Department of Medical Sciences and Public Health, University of Cagliari, Cagliari, Italy; 3 Department of Neuroscience, University of Turin, Turin, Italy; 4 Department of Psychology, University of Cagliari, Cagliari, Italy; 5 Azienda Regionale della Salute (ARES, Sardegna), Medio Campidano, Italy; 6 University of Cagliari, Cagliari, Italy

**Keywords:** Questionnaire, Adaptive hyperactivity, Goal achievement, Factorial validity, Older adults, AHGA

## Abstract

**Objective:**

This paper illustrates the preliminary psychometric properties of the Questionnaire for Adaptive Hyperactivity and Goal Achievement (AHGA), aimed at measuring adaptive characteristics of hyperactivity and goal pursuit in older adults.

**Methods:**

The 12-item scale was administered to a sample of 120 subjects (older adults) between February 2022 and June 2022. The reliability of AHGA was measured using Cronbach’s alpha, and factor structure was established using parallel analysis (PA) and principal component analysis (PCA). Convergent validity was tested against the Biological Rhythms Interview of Assessment in Neuropsychiatry (BRIAN).

**Results:**

All included subjects have an average age of 74.1±5.1 years. AHGA reliability was good (Cronbach’s alpha: 0.713 [95%CI: 0.630 to 0.783]). Factor analysis suggested two main components: goal achievement and hyperactivity, which explained 41% of the variance in the data. The results support the convergent validity of the scale: AHGA measures adaptive characteristics of hyperactivity and goal pursuit, in contrast to BRIAN, which measures pathological characteristics.

**Conclusion:**

The reported findings represent an innovative approach to hyperthymic features by embracing a broader spectrum concept that conceptualizes the potential transition between pathological and adaptive aspects as a continuum.

## 1 INTRODUCTION

Modern city life can have an impact on mental health. Indeed, psychiatric disorders tend to be more frequent in urban areas, and their onset, severity, and prognosis are influenced by their exposure to urban environments [1]. The urban-specific characteristic and consequent adaption have a high level of vaiability and can mediate for a variety of environmental influences [2]. Several factors can affect the regularity of biological rhythms as a part of modern city life, exposing people to strong stress but also constituting a potential advantage in terms of adaptation under peculiar circumstances. This potentially adaptive effect might be more evident in the megacities, in which life is supposed to occur 24 hours a day, seven days a week, with complete alterations of sleep-wake cycles [3, 4]. From this point of view, the presence of genes and genetic variants that are associated with a high risk of bipolar disorder (CACNA1C, ANK3, NCAN, TRANK1, TENM4, and SYNE1) in the population may be related to adaptive characteristics in particular circumstances [5, 6]. On one side, they can therefore precede success in relation to the performance that a contemporary urban lifestyle often demands, or, on the other side, they can predispose to the risk of developing mood disorders or bipolar disorders. Cornerstones, such as creativity, extraversion, leadership, and openness can spread from adaptive and functional utility to constitute a vulnerability to mental illnesses in specific adverse conditions [7]. From an epidemiological perspective, the hypothesis of adaptive aspects of hyperactivity may explain certain phenomena in modern societies, as witnessed by the continuing increase in the prevalence of mood disorders [3]. Behaviours, responses, and reactions as responses to specific demands of the environment might resemble bipolar spectrum disorders, but this could be just a form of adaptive behaviour [8]. This could justify the growing number of overdiagnoses of bipolar disorder [9]. Moreover, some adaptive features of hyperactivity may drive people to novelty-seeking, such as explorers with hyperthymic temperaments and migrants [10].

Тhe, two basic components of this type of temperament that stand out, are hyperactivity and goal achievement. As previously described, a hyperthymic temperament manifests extroversion, a high energy level, emotional intensity, and a moderate need for sleep. Actually, these kinds of traits seem to provide well-defined advantages in leadership, competition, exploration, and territoriality. The hyperthymic temperament can be classified as abnormal only in the presence of chronic hypomanic symptoms or advanced mood disorders [11, 12]. In general, temperaments have been verified to belong to the domain of normality rather than the sphere of pathology in accordance with their putative adaptive role.

Commonly, achieving success, experiencing excitement and joy, and moving towards core life goals are moments of great importance in life. Under this approach, goal and drive achievement display an adaptive and beneficial side of hyperthymia. On the positive side, setting high goals is one of the strongest predictors of success. It indicates the willingness to set high goals and spend energy pursuing them, which could help clarify the high rates of creative efforts among people with hyperthymic temperament. However, the pathological scenery appears linked to variability in the adaptiveness with which people follow life goals and accomplishments [13, 14].

Within this framework, this paper proposes a new questionnaire to measure some adaptive characteristics of hyperactivity and goal pursuit concerning the notion that emotional dysregulation is a marker of the passage to pathology in older adults [15].

This paper illustrates the results of administering the Questionnaire for Adaptive Hyperactivity and Goal Achievement (AHGA) aimed at providing preliminary evidence on its reliability, factor structure, and capacity in older adults.

## 2 METHODS

### 2.1 Description of the Sample

The investigation of the reliability and factor structure of the AHGA was carried out through its administration to a sample of 120 older adults selected for a previous randomized controlled trial (RCT) [16, 17], according to the Declaration of Helsinki and its revisions [18]. The Ethical Committee of the Institutional Review Board of the University Hospital of Cagliari, Italy, approved this RCT, which included subjects who provided written informed consent [16, 17]. Sample had previously been recruited by the Department of Medical Science and Public Health, University of Cagliari, Italy. The following inclusion criteria were implemented: elderly people aged 65 or older of both genders, living at home, and being able to provide informed consent without impairment for severe diseases (*e.g*., mild cases of hypertension or diabetes were admitted). Additional inclusion criteria included the absence of a lifetime history of diagnosed bipolar spectrum disorder conditions.

Exclusion criteria were: refusal to participate in the study (non-signing the informed consent for accepting participation in the study); participants with a diagnosis of bipolar spectrum disorder, and participants under 65 years of age. All included subjects provided written informed consent and were invited to fill out the Italian version of the Questionnaire for Adaptive Hyperactivity and Goal Achievement (AHGA).

### 2.2 Measures

Socio-demographic information was collected for the following variables: age, sex, socioeconomic status, and civil status. Measures of socioeconomic status were grouped into four categories: elementary school diploma, lower secondary diploma, upper secondary diploma, and university degree [19].

AHGA is a 12-item, five-point Likert scale (from 1 to 5), developed by a team of PhD students, psychotherapists, and psychiatrists with decades of expertise in bipolar spectrum disorders from the University of Cagliari. The final version was reached through a process of item development and selection followed by administering the tool to the same target population of subjects not directly enrolled in the validation phase. This stage was carried out carefully to identify potential issues or unclear terms or items in order to improve the quality and understandability of the questionnaire.

The questions mainly focus on two areas: lifelong adaptive hyperactivity and goal achievement. The tool investigates transfers, employment differences, and economic well-being compared to parents, as well as the perceived speed in doing things and thoughts flow. Other elements of the investigation are related to the usual number of people with whom they have conversations, the amount of weekly physical activity, the number of hours spent outside the home and the number of user applications or programs during the day. Elements such as how many times a person has been in love in their lifetime, without even being in an official relationship, along with the average number of hours slept during the night, are also AHGA components. The original Italian version is included as an appendix in the current paper.

Since this questionnaire has a role in measuring principally adaptive hyperactivity, the Biological Rhythms Interview of Assessment in Neuropsychiatry (BRIAN) serves to indicate particularly pathological hyperactivity and dysregulation of biorhythms, predisposing factors that could suggest the presence of a certain psychiatric disorder.

BRIAN is an 18-item interviewer administered-instrument that focuses on evaluating four main areas related to circadian rhythm disturbance: sleep, activities, social rhythms, and eating patterns. Items are estimated using a 4-point scale: (1)=no difficulty, (2)=mild difficulty, (3)=moderate difficulty, and (4)=severe difficulty. The BRIAN scores, therefore range from 1 to 72, where higher scores indicate serious circadian rhythm disturbances [20].

### 2.3 Statistics

Data were imputed in Excel and analysed using the Statistical Package for Social Sciences (SPSS) version 27. Additional analyses were carried out in R. All tests were two-tailed, with alpha set at p<0.05.

Means with standard deviation counts and percentages were used to describe the distribution of the data in the sample. An ANOVA, when appropriate, was used to determine whether continuous variables differed among groups of subjects. The Games-Howell test was used to test post-hoc differences in groups since it does not assume equal variances and sample size. Chi-square tests were used for categorical data. Pearson’s correlation coefficient was used for correlations. Reliability was measured as internal coherence using Cronbach’s alpha. For group comparisons, a rule of thumb assumes that reliability values of 0.70 are considered acceptable [21].

The data was preliminarily subjected to a principal component analysis (PCA) to establish the spontaneous distribution of the 12 core items of the AHGA into one or more separate dimensions. Parallel analysis was used to determine the optimal number of components. In a parallel analysis, the scree plot of the observed data was compared with that of a random matrix of the same size as the original. The best solution is based on the number of components with eigenvalues higher than those generated by the random data, either simulated or reassembled by permutation from the original data. The parallel analysis and the subsequent PCA were carried out with the psych package running in R [22, 23]. Both were applied to a matrix of polychoric correlations since the data were ordinal.

After establishing the factor structure of the Italian version of AHGA, the correlation of its measures with those of BRIAN was assessed to check the convergent validity of the scale and its factors.

## 3 RESULTS

The sample was balanced by age and gender (Table **1**). There was a slight excess of married people among men and widowed people among women (Chi-square = 10.4; df=3; p = 0.015).

### 3.1 Reliability

The reliability of the 12-item AHGA, measured as internal consistency, was good: Cronbach’s alpha in the sample was 0.713 (95%CI: 0.630 to 0.783).

### 3.2 Factor Analysis and Extraction of the Main Components

**Fig. (1):**
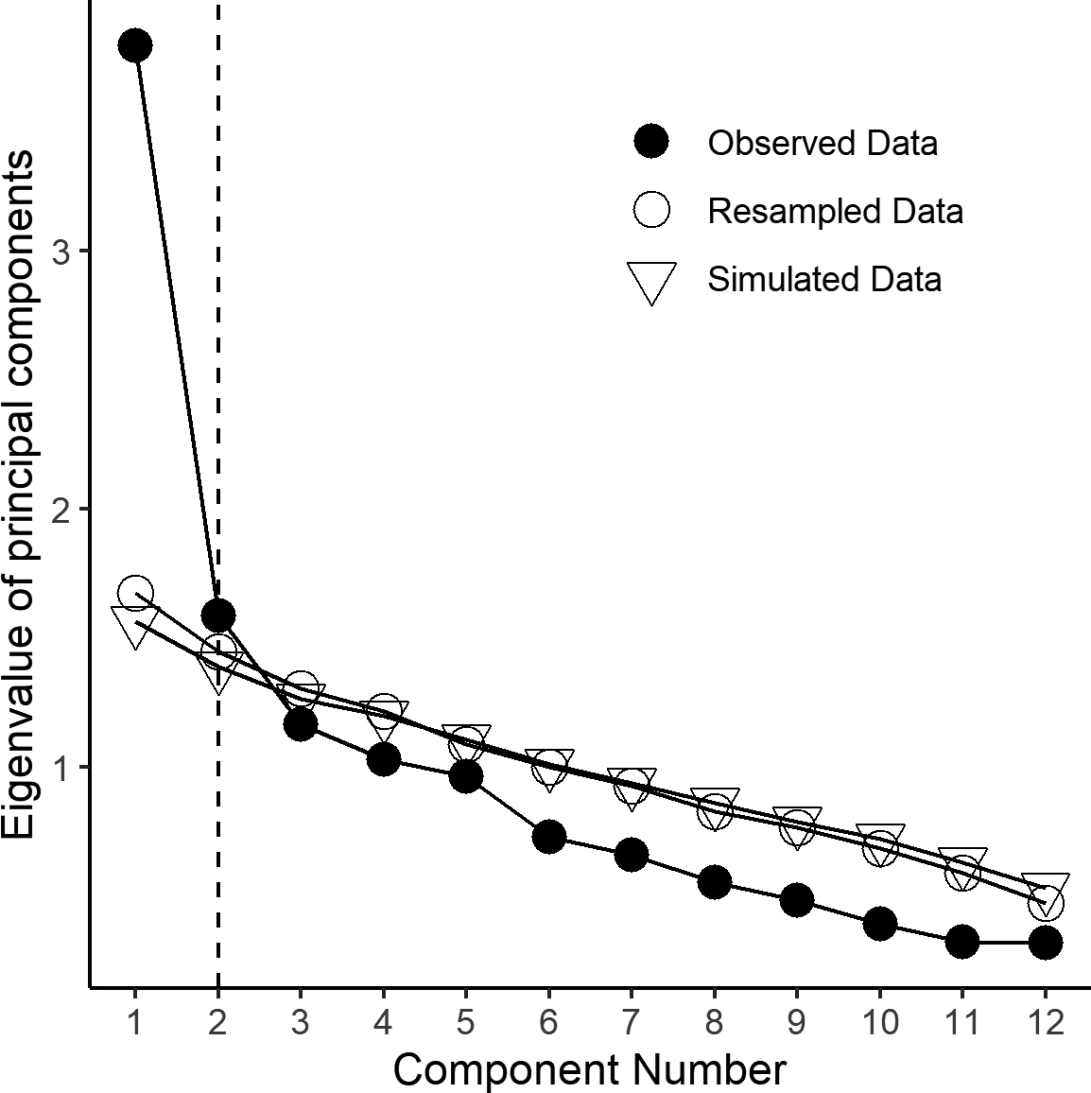
Parallel analysis was applied to the items of the Questionnaire for Adaptive Hyperactivity and Goal Achievement (n=12). A plot of the eigenvalues calculated on the basis of the actual data and the simulated and resampled data.

Bartlett’s test of sphericity was 288.7542 (p < 0.0001), Kaiser-Meyer-Olkin’s adequacy value was 0.78. The matrix, thus, can be factorized (Fig. **1**).

The parallel analysis suggested a total of two principal components (Fig. **1**). The main components were then analyzed by estimating two factors (Table **2**).

**Fig. (2):**
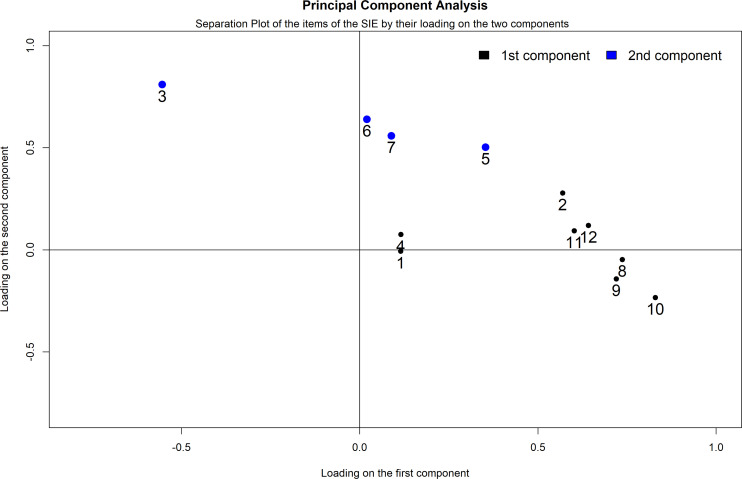
A separation plot of the results of the principal component analysis is applied to the items of the Questionnaire for Adaptive Hyperactivity and Goal Achievement (n=12). Items are plotted on the basis of their loadings on the two extracted main dimensions.

The separation of the two factors with the varimax technique did not differ from the separation of the two factors with the Promax technique, which admits correlation between the two factors as probable based on their nature. Based on the direction of loading (assigned to the item only if loading is positive) and the relative weight, items 2, 8, 9, 10, 11, and 12 were assigned to the first factor or first component (goal achievement), while items 3, 5, 6, and 7 were assigned to the second factor or second component (hyperactivity), while items 1 and 4 were not clearly separated between the two factors (the two components) (Fig. **2**).

Subscale 1, comprising items 2, 8, 9, 10, 11, and 12, was named “goal achievement” based on the thematic content items. Subscale 2, comprising items 3, 5, 6, and 7, was named “hyperactivity” based on the thematic content of the items. The score of the two subscales for each participant was obtained by adding the values of each item and dividing by the number of items to obtain a homogeneous interval from 1 to 5, with scores increasing as the expression of the variable measured by the subscale increases.

Fig. (**3**) summarizes the item distribution in the two components, as well as the variance fraction. The solution explained 41% of the total variance.

### 3.3 Relationship of the Main Components of the AHGA with Socio-demographic Indicators

The distribution of the score concerning subscale 1 (goal achievement) did not differ among men (mean ± SD: 3.6 ± 0.8) and women (3.4 ± 0.8); F[2;116]=, 683; p = 0,057. On the other hand, subscale 2 (hyperactivity) showed higher scores among men (3.1 ± 0.7) than women (2.8 ± 0.9); F[2;116]=,039; p = 0.014.

No relationship was observed in the distribution of the two subscales in relation to age: subscale 1 (Pearson r = 0.081; p = 0.380) and subscale 2 (Pearson r = 0.063; p = 0.493). Also, no difference was observed in the distribution of the scores of the two subscales concerning the civil status. Instead, there was observed an increase in the scores of the two subscales equivalent to the growth of the level of education. In particular, subscale 1 showed higher scores among those who had obtained upper secondary and university diplomas than those with lower educational levels. The subjects with university diploma seem to be more goal achievers, perhaps partly reflecting the chances/resources, but also the type of question (there is a question about the school level and one about income, which comes partly from the educational level) (Table **3a**-**3c**).

### 3.4 Concurrent and Divergent Validity Analyses

We investigated the links between adaptive hyperactivity and goal achievement scale and a measure of the rhythms in bipolar disorder, the BRIAN. The analysis was done with Pearson’s correlation coefficient (r), using a threshold of p<0.05 for statistical significance. We found that the goal achievement subscale of the scale (factor 1) was negatively related to the BRIAN and its subscales, except for the rhythm (Table **4**).

The hyperactivity subscale (factor 2) was less negatively related to the BRIAN and its subscales (Table **4**). Essentially, greater propensity to goal achievement was related to greater disruption of sleep, general activity, sociality, and eating habits. Greater propensity to hyperactivity only disrupted general activity and sociality. The results support the convergent validity of this new scale, since the higher the propensity to display symptoms of bipolar disorder, as higher the impact on social rhythms.

## 4 DISCUSSION

From an evolutionary point of view, there are many examples that describe conditions that are “maladaptive” in a certain environment but can become adaptive as the context conditions change [24]. The best-known case of such a phenomenon is the evolution of the peppered moth. During the Industrial Revolution, the common white “bladder” peppered moth was replaced by a black “carbonara” type of specimen. The interaction between bird predation and coal pollution drove this evolution. Whenever an apparently maladaptive condition inexplicably appears and grows, a similar phenomenon can be found [25]. In humans, some lifestyle circumstances accompanied by the possible dysregulation of biological rhythms, for example those of the urban lifestyle, play a crucial role as triggers of bipolar spectrum disorders; however, hyperactivity and goal pursuit is actually the main drivers of adaptive behavior in specific living conditions. This could explain the increasing trend in diagnoses of bipolar spectrum disorders in modern societies [3, 26]. Hence, it cannot be excluded that some hypomanic characteristics may be adaptive in some social contexts [27].

Nowadays it is widely debated whether affective temperaments, including hyperthymia, belong to the area of pathology or to that of normality. Hyperthymic temperament is characterized by high levels of emotions and feelings and the ability to connect to people. This kind of temperament appeared to enhance emotion and satisfaction in life, mental health, and social support [28-30]. This quality is adaptively advantageous when such persons are approached by illness, suffering, or death, which is unavoidable with advancing age. As well, several studies claimed that hyperthymic temperament prevents suicidal ideas and efforts [31-33]. In an Argentinian study, the quality of life of the hyperthermic individuals was equal to that of controls, qualifying hyperthymia as one of the most adaptive temperamental types in some circumstances and environments [34]. Regarding hyperactivity, males showed a greater inclination towards this trait. Hyperactivity plays a major role in reducing the reactivity to life stressors, and expanding a significant ability to relate to others [35].

As presented in the results, higher scores in goal achievement were observed among those who had achieved the highest levels of education. Goal achievers develop an intense sense of competition and a strong focus on accomplishments, which are valued above rules and circumstances [36, 37].

The existential dimension in which the challenge, overcoming boundaries and living emotionally intense experiences are a priority seems to be an integral part of this temperamental trait of living. Particularly, individuals with this trait actively accept conflicts with their surroundings rather than follow the prevalent public belief [28, 38]. From a pathological view, people with bipolar disorders report being particularly perfectionists in pursuing goals, and seeing goal accomplishment as more important to their self-worth perception [39].

Another key aspect of our study was the sample features, given that the sample is made up of people aged 65 and over without any lifetime psychiatric condition, it is considered quite stable in terms of the possible onset of psychiatric disorders typical of other ages [40, 41]. In fact, due to the pathoplastic effect of age, the older the interviewee gets, the greater the chances of cumulative life events. The seemingly constant number of possible “events” after a certain age represents a predictable scenario.

The preliminary nature of the data represents an obvious limitation of the study; it will be necessary to replicate them to a greater extent. Another limitation lies in the characteristics of the sample. As mentioned above, participants mainly come from the previous study, where different physical and cultural activities are carried out. This factor could define a bias in the sample definition phase, given the presence of a higher frequency of hyperthymic features among the included participants. Further studies, including samples of more diverse socio-cultural and ethnic populations, are needed for a comprehensive understanding of the factorial structure of the AHGA.

## CONCLUSION

The results support the convergent validity of the Questionnaire for Adaptive Hyperactivity and Goal Achievement (AHGA), which measures adaptive characteristics of hyperactivity and goal pursuit in contrast to BRIAN, which measures pathological characteristics. The results represent the first step in an innovative approach aimed at integrating adaptive and pathological aspects of hyperthymic features by embracing a broader concept of the spectrum that conceptualizes these elements as intrinsically linked.

## AUTHORS' CONTRIBUTIONS

GK has prepared the first draft, AP prepared the statistical analysis, GC, MGC revised it. All authors, GK, GC, AP, MTL, SF, DP, RZ, VM, ME, EP, MGC, revised and approved the final version of the paper.

## Figures and Tables

**Fig. (3):**
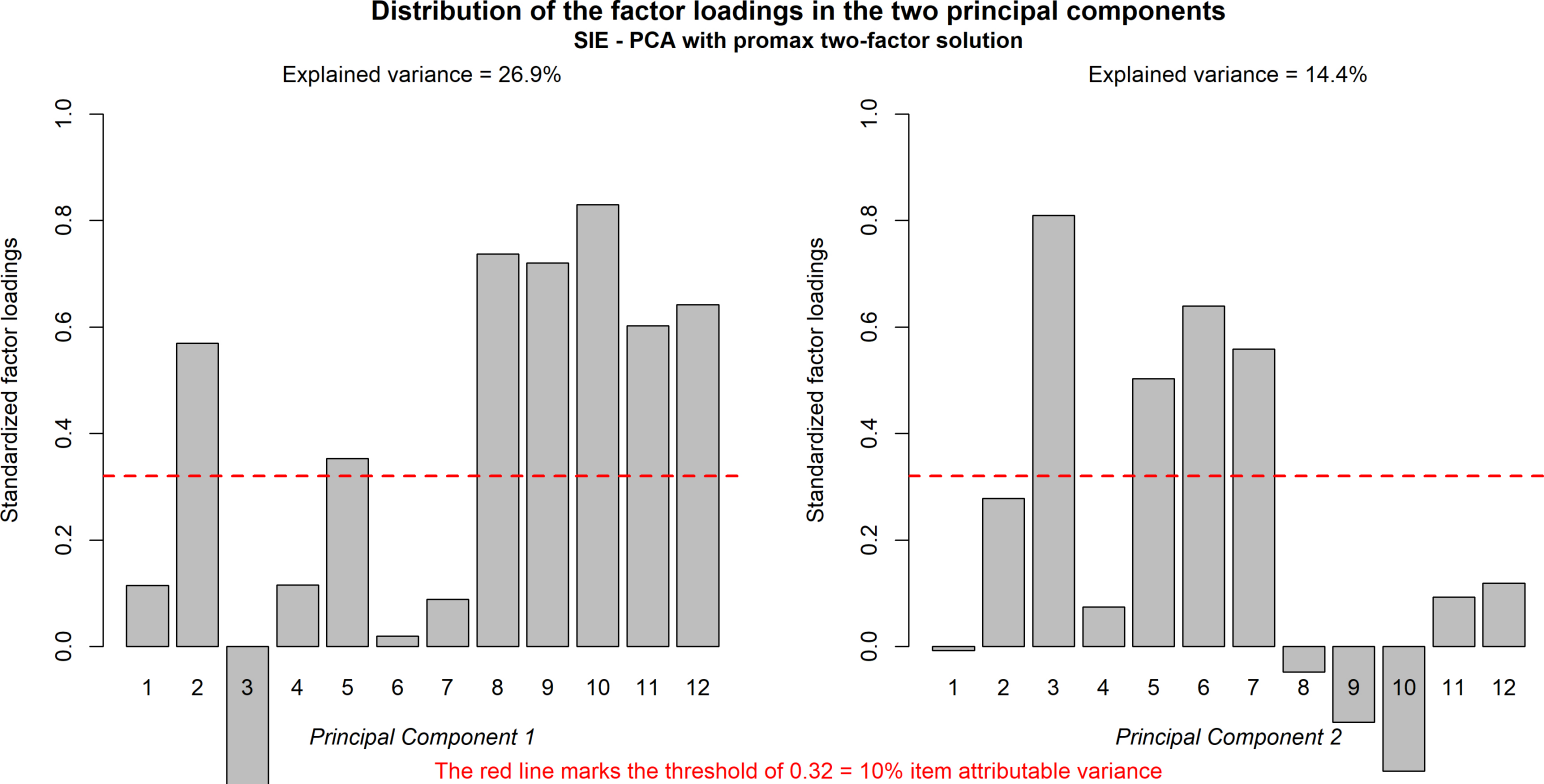
Distribution of the factor loadings in the two principal components.

**Table 1:** General epidemiological characteristics of the sample (n=120).

-	Men	Women	Total
Gender	56 (47%)	64 (53%)	120 (100%)
Age	74.5 (5.2)	73.8 (5.1)	74.1 (5.1)
Education level Elementary school Lower secondary Upper secondary University	5 (9%) 10 (18%) 23 (41%) 18 (32%)	4 (6%) 13 (20%) 27 (42%) 20 (31%)	9 (7%) 23 (19%) 50 (42%) 38 (32%)
Civil Status Unmarried/non cohabiting Married/cohabiting Widower/widow Non specified	7 (12%) 42 (75%) 6 (11%) 1 (2%)	13 (20%) 32 (50%) 19 (30%) 0 (0%)	20 (17%) 74 (61%) 25 (21%) 1 (1%)

**Table 2:** Principal component analysis (PCA) of the Questionnaire for Adaptive Hyperactivity and Goal Achievement (n=120).

**Item**	**First component (goal achievement)**	**Second component (hyperactivity)**
1	0.11	-0.01
2	0.57	0.28
3	-0.55	0.81
4	0.12	0.07
5	0.35	0.50
6	0.02	0.64
7	0.09	0.56
8	0.74	-0.05
9	0.72	-0.14
10	0.83	-0.23
11	0.60	0.09
12	0.64	0.12
SS loadings	3.23	1.73
Proportion of variance	0.27	0.14
Cumulative variance	0.27	0.41

**note:** SS (squared loadings)

**Table 3a:** Descriptive analysis (educational level).

Descriptive Analysis (Educational Level)									
-	-	-	-	-	-	95% Confidence Interval for the Mean		-	-
-		Number	Mean	Standard Deviation	Standard Error	Lower Limit	Upper Limit	Minimum	Maximum
Factor 1 (goal achievement)	2 (Elementary School)	9	2,370	,7807	,2602	1,770	2,971	1,3	3,8
	3 (Lower Secondary)	23	3,043	,6709	,1399	2,753	3,334	1,8	4,0
	4 (Upper Secondary)	50	3,860	,6532	,0924	3,674	4,046	2,2	4,8
	5 (University)	38	3,632	,7535	,1222	3,384	3,879	2,0	5,0
	Total	120	3,519	,8203	,0749	3,371	3,668	1,3	5,0
Factor 2 (hyperactivity)	2 (Elementary School)	9	2,472	,7009	,2336	1,933	3,011	1,5	3,3
	3 (Lower Secondary)	23	2,804	,8981	,1873	2,416	3,193	1,5	5,0
	4 (Upper Secondary)	50	3,060	,7963	,1126	2,834	3,286	1,0	4,8
	5 (University)	38	3,046	,8888	,1442	2,754	3,338	1,0	4,8
	Total	120	2,963	,8474	,0774	2,809	3,116	1,0	5,0

**Table 3b:** ANOVA analysis (educational level).

ANOVA (Educational Level)						
-	-	Sum of Squares	df	Quadratic Mean	F	Sig.
Factor 1 (Goal Achievement)	Between the groups	23,371	3	7,790	15,939	<,001
	Within the groups	56,695	116	,489	-	-
	Total	80,066	119	-	-	-
Factor 2 (Hyperactivity)	Between the groups	3,479	3	1,160	1,641	,184
	Within the groups	81,977	116	,707	-	-
	Total	85,456	119	-	-	-

**Table 3c:** Multiple comparisons regarding the level of education.

Multiple Comparisons							
Games-Howell							
-	-	-	-	-	-	95% confidence interval	
Dependent variable	(I) Level of Education	(J) Level of Education	Difference of the Mean (I-J)	Standard Error	Sig.	Lower Limit	Upper Limit
Factor 1 (Goal Achievement)	2 (Elementary School)	3 (Lower Secondary)	-,6371	,2955	,154	-1,541	,195
		4 (Upper Secondary)	-1,4896*	,2762	,001	-2,333	-,647
		5 (University)	-1,2612*	,2875	,004	-2,117	-,405
	3 (Lower Secondary)	2 (Elementary School)	,6731	,2955	,154	-,195	1,541
		4 (Upper Secondary)	-,8165*	,1676	<,001	-1,265	-,368
		5 (University)	-,5881*	,1858	,013	-1,082	-,095
	4 (Upper Secondary)	2 (Elementary School)	1,4896*	,2762	,001	,647	2,333
		3 (Lower Secondary)	,8165*	,1676	<,001	,368	1,265
		5 (University)	,2284	,1532	,448	-,174	,631
	5 (University)	2 (Elementary School)	1,2612*	,2875	,004	,405	2,117
		3 (Lower Secondary)	,5881*	,1858	,013	,095	1,082
		4(Upper Secondary)	-,2284	,1532	,448	-,631	,174
Factor 2 (Hyperactivity)	2 (Elementary School)	3 (Lower Secondary)	-,3321	,2994	,688	-1,175	,511
		4 (Upper Secondary)	-,5878	,2594	,161	-1,357	,182
		5 (University)	-,5738	,2746	,201	-1,368	,219
	3 (Lower Secondary)	2 (Elementary School)	,3321	,2994	,688	-,511	1,175
		4 (Upper Secondary)	-,2557	,2185	,649	-,842	,331
		5 (University)	-,2417	,2363	,737	-,872	,388
	4 (Upper Secondary)	2 (Elementary School)	,5878	,2594	,161	-,182	1,357
		3 (Lower Secondary)	,2557	,2185	,649	-,331	,842
		5 (University)	,0139	,1830	1,000	-,467	,495
	5 (University)	2 (Elementary School)	,5738	,2746	,201	-,219	1,366
		3 (Lower Secondary)	,2417	,2363	,737	-,389	,872
		4 (Upper Secondary)	-,0139	,1830	1.000	-,495	,467

Note:*The difference in the mean is significant at the level of 0.05

**Table 4:** Correlation analysis between the Adaptive Hyperactivity and Goal Achievement scale and the brian.

-	Factor 1 Goal achievement	Factor 2 Hyperactivity
BRIAN (Total)	r = -0.368; p<0.0001	r = -0.208; p = 0.040
Sleep	r = -0.285; p = 0.004	r = -0.153; p = 0.132
Activity	r = -0.273; p = 0.007	r = -0.221; p = 0.029
Sociality	r = -0.246; p = 0.015	r = -0.208; p = 0.040
Eating	r = -0.255; p = 0.011	r = -0.066; p = 0.519
Rhythm	r = -0.101; p = 0.321	r = -0.116; p = 0.257

## Data Availability

The dataset that supports the results and findings of this research are available from the corresponding author upon reasonable request.

## LIST OF ABBREVIATIONS

AHGA: = Adaptive Hyperactivity and Goal Achievement
PA: = Parallel Analysis
PCA: = Principal Component Analysis
BRIAN: = Biological Rhythms Interview of Assessment in Neuropsychiatry

## APPENDIX

Questionario sull'iperattività adattiva e sulla spinta all'esplorazione (Italian-original version)

Età:

Genere:

- Maschio
- Femmina
- Preferisco non specificare

Livello di istruzione:

- Non ho frequentato la scuola
- Diploma di Scuola elementare
- Diploma di Scuola media
- Diploma di Scuola superiore
- Laurea

1. Quante volte pensi di esserti innamorato/a (anche senza avere una relazione)?

- Tra Nessuna e 1 volta
- Tra 2 e 3 volte
- Tra 4 e 5 volte
- Tra 6 e 7 volte
- Più di 7 volte

2. Quante volte ti sei spostato in un raggio oltre i 200 km dal luogo in cui vivevi?

- Meno di 3
- Tra 3 e 5
- Tra 6 e 8
- Tra 9 e 11
- Più di 11

3. In media, con quante persone fai una chiacchierata in una settimana?

- Meno di 5
- Tra 5 e 10
- Tra 11 e 15
- Tra 16 e 20
- Più di 20

4. Quante ore dormi in media ogni notte?

- Meno di 4
- Tra 4 e 5
- Tra 6 e 7
- Tra 8 e 9
- Più di 9

5. Quante ore dedichi a settimana all’attività fisica? (es. camminata veloce, tennis, corsa, o sport in generale, attività in campagna, faccende domestiche, ballo)

- Meno di 1
- Tra 1 e 2
- Tra 3 e 4
- Tra 5 e 6
- Più di 6

6. Quante ore al giorno passi fuori casa?

- Meno di 1
- Tra 1 e 2
- Tra 3 e 4
- Tra 5 e 6
- Più di 6

7. Quanti programmi/applicazioni usi al giorno? (ad esempio: Posta Elettronica, Skype, Netflix, Facebook, WhatsApp)

- Tra 0 e 1
- 2
- 3
- 4
- 5 o più

8. Quanto è diverso il lavoro che hai fatto rispetto a quello del genitore del tuo stesso sesso?

- Per nulla
- Poco
- Abbastanza
- Molto
- Moltissimo

9. Il tuo livello scolastico, rispetto a quello del genitore con il livello più alto è:

- Inferiore
- All’incirca uguale (più o meno un anno scolastico di differenza)
- Un poco maggiore (dai due ai cinque anni di differenza)
- Maggiore (dai sei agli otto anni di differenza)
- Di gran lunga superiore (oltre otto anni di differenza)

10. Come valuti il tuo benessere economico/reddito rispetto a quello dei tuoi genitori:

- Inferiore
- All’incirca uguale (tenuto conto anche delle differenze di epoca)
- Un poco maggiore
- Maggiore
- Di gran lunga superiore

11. Mi sembra che gli altri siano:

- Molto più veloci di me nel fare le cose
- Più veloci di me nel fare le cose
- Abbiano la mia stessa velocità nel fare le cose
- Io sia più veloce degli altri nel fare le cose
- Io sia molto più veloce degli altri nel fare le cose

12. Mi sembra che gli altri siano:

- Molto più veloci di me nel pensare
- Più veloci di me nel pensare
- Abbiano la mia stessa velocità nel pensare
- Io sia più veloce degli altri nel pensare
- Io sia molto più veloce degli altri nel pensare
